# Parental Competence in Parents of Children with Autism Spectrum Disorder: A Systematic Review

**DOI:** 10.17533/udea.iee.v37n3e03

**Published:** 2019-10-23

**Authors:** Fatemeh Mohammadi, Mahnaz Rakhshan, Zahra Molazem, Mark Gillespie

**Affiliations:** 1 RN, Ph.D. Assistant professor, School of Nursing and Midwifery, Chronic Diseases (Home Care) Research Center, Hamadan University of Medical Sciences, Hamadan, Iran: mohammadifateme47@yahoo.com Hamadan University of Medical Sciences Hamadan Iran mohammadifateme47@yahoo.com; 2 RN, Ph.D. Associate professor, School of Nursing and Midwifery, Shiraz University of Medical Sciences, Shiraz, Iran. Email: mzrakhshan@gmail.com(Corresponding Author). Shiraz University of Medical Sciences Shiraz Iran mzrakhshan@gmail.com; 3 RN, Ph.D. Associate professor, School of Nursing and Midwifery, Shiraz University of Medical Sciences, Shiraz, Iran. Email: molazem@sums.ac.ir Shiraz University of Medical Sciences Shiraz Iran molazem@sums.ac.ir; 4 MEd, Nursing Lecturer. School of Health Nursing and Midwifery, University of the West of Scotland, Paisley, Scotland, United Kingdom. Email: Mark.Gillespie@uws.ac.uk University of the West of Scotland University of the West of Scotland Paisley United Kingdom Mark.Gillespie@uws.ac.uk

**Keywords:** autistic disorder, child, parents, systematic review., trastorno autístico, niño, padres, revisión sistemática., transtorno autístico, criança, país, revisão sistemática.

## Abstract

**Objective.:**

This work aimed to define and assess the parental competence of parents with autistic children.

**Methods.:**

This study was conducted through a systematic review. The search was done in databases, including Cochrane Library, PubMed, CINAHL, Science Direct, Wiley Scopus, Pro Quest, Web of Science, Elsevier, Google Scholar, and Ovid by using keywords, like “children, autism, parenting, competence, and scale” from 1974 to 2019. Inclusion criteria were that the article should be quantitative, qualitative, and mixed-method studies in nursing, psychology, and medicine; the full text of the article should be available and the article should be in English or Persian.

**Results.:**

Competence among these parents was affected by more factors and they reported lower competence compared to other parents. Moreover, only two instruments were available to assess parenting competence, which were not designed for parents of autistic children. Variables and factors affecting parenting competence has not been examined well in parents of children with autism, and no specialized instrument is available to evaluate parenting competence in parents with autistic children either.

**Conclusion.:**

Although parental competence has been known as the main element to improve the quality of care, it has been studied restrictively from the viewpoints of the parents of children with autism. Therefore, the development of this concept is highly essential for clinical application and investigating its outcomes support.

## Resumen

**Objetivo.:**

Evaluar la competencia parental de los padres con hijos autistas.

**Métodos.:**

Revisión sistemática narrativa. La búsqueda se realizó en las bases de datos de la Biblioteca Cochrane, PubMed, CINAHL, Science Direct, Wiley Scopus, ProQuest, Web of Science, Elsevier, Google Scholar y Ovid, mediante el uso de las palabras clave "niños, autismo, crianza, competencia y escala". Los criterios de inclusión fueron: artículos originales o de revisión publicados entre 1974 a 2019; estudios cuantitativos, cualitativos o de métodos mixtos en las disciplinas de enfermería, psicología o medicina, y estar disponible en texto completo en inglés**.**

**Resultados.:**

La competencia de los padres de niños con trastorno del espectro autista fue menor en comparación con padres de niños sin este trastorno. Existen dos escalas para la evaluación de la competencia parental, pero no se diseñaron para su evaluación en padres de niños autistas. Los factores que afectan la competencia parental tampoco se han examinado exclusivamente para estos padres**.**

**Conclusión.:**

Aunque se sabe que la competencia de los padres es un elemento primordial para mejorar la calidad del cuidado del niño con autismo, el tema se ha estudiado de manera restrictiva. Es necesario el desarrollo de este concepto para la aplicación en la práctica clínica y para la investigación de sus resultados.

## Resumo

**Objetivo.:**

Avaliar a competência parental dos pais com filhos autistas.

**Métodos.:**

Revisão sistemática narrativa. A busca se realizou nas bases de dados da Biblioteca Cochrane, PubMed, CINAHL, Science Direct, Wiley Scopus, ProQuest, Web of Science, Elsevier, Google Scholar e Ovid, mediante o uso das palavras clave "crianças, autismo, criança, competência e escala". Os critérios de inclusão foram: artigos originais ou de revisão, publicados entre 1974 a 2019, ser estudos quantitativos, qualitativos ou de métodos mistos, nas disciplinas de enfermagem, psicologia ou medicina, e estar disponível em texto completo em inglês**.**

**Resultados.:**

A competência dos pais de crianças com transtorno do espectro autista foi menor em comparação com pais de crianças sem este transtorno. Existem duas escalas para a avaliação da competência parental, mas não foram desenhadas para sua avaliação em pais de crianças autistas. Os fatores que afetam a competência parental tampouco se hão examinado exclusivamente para estes pais**.**

**Conclusão.:**

Embora se sabe que a competência dos pais é um elemento primordial para melhorar a qualidade do cuidado da criança com autismo, o tema se há estudado de maneira restritiva. É necessário o desenvolvimento deste conceito para a aplicação na prática clínica e para a investigação de sus resultados.

## Introduction

Autism is one of the most important developmental disorders, which severely affects both parents and children.(1) This disorder imposes great physical, mental, and social pressures on families, particularly parents,(2) and affects various dimensions of their lives.(3) These parents, especially mothers, try to adapt with the painful events and the care burden and seek support from various resources.(4) Hence, the healthcare providing is required to identify the needs and problems of these parents and help them to do their parenting roles well.(5) In order to identify the problems of parents of children with autism, in recent years many studies have focused on the assessment of stress level and compatibility strategies in parents of autistic children.(6) These studies, in fact, aimed to find strategies for reducing the impacts of these children on other family members, particularly mothers(7) The results of these studies indicated that such mothers had lower health status, higher levels of stress, and lower self-efficacy and parenting competence compared to mothers with healthy children as well as those with children suffering from other disorders.(8,9) Although some studies have examined parental self-efficacy among parents of children with autism, parental competence is more comprehensive and broader than parental self-efficacy.(10) Some studies have attempted to define the concept of parental competence in parents of children with normal growth and development,(11,12) however the systematic review revealed that this concept is not explained in the parents of children with autism; when parents face their children’s severe conditions, their competence will be significantly affected by their children’s sickness and other background factors.(13,14) Also according to several studies, parental competence in these parents are influenced by more factors than the normal children's parents.(13-15)

It is important that nurse’s awareness of the effective factors in parenting competence among parents of autistic children that they can take basic steps in identifying the problems of these parents, promoting parenting competence and subsequently promote the quality of life of parents and the child with autism. In addition, definition, dimensions, and effective factors of parenting competence are not established well in such parents. The aim of the present study is to answer the question, which is to define and assess the parental competence of the parents with autistic children? 

## Methods

The present study is a comprehensive systematic review of the available literature on parental competence among parents of autistic children. The applied approach to systematic review is based on Cochrane's guideline which consists of topic selection, inclusion criteria, search strategy, selection of studies, evaluation of the quality of articles, data collection, analysis and conclusion.(16) The entire articles related to parental competence were published over the past forty years, from 1974 to 2019, and were checked electronically. The collected resources based on publication year, country of origin, type, research type and domains have been summarized in Table 1.

**Table 1:** Summary of the collected resources

Variable	***n* (%)**
Year of Publication	
1974-2000	11 (24.44)
2001-2010	27 (60.00)
2011-2019	7 (15.56)
Country of origin	
Iran	5 (11.11)
United States	11 (24.44)
Canada	7 (15.56)
United Kingdom	9 (20.00)
Australia / New Zealand	6 (13.33)
Others	7 (15.56)
Reference type	
Best practice implementation article	31 (68.88)
News brief	8 (17.78)
Commentary/letter to editor/editorial	5 (11.11)
Book/book review	1 (2.23)
Domain	
Clinical	35 (77.78)
Educational	6 (13.33)
Theoretical/conceptual	4 (8.89)
Research type	
Quantitative	30 (66.67)
Qualitative	10 (22.22)
Mixed	5 (11.11)

Inclusion criteria. The articles related to parental competence published between 1974 and 2017 were examined by the researchers. The following criteria were used for inclusion: 1) The article should be original and have the qualities of a review study; 2) The full text of the article should be available; 3) The article should be in English or Persian; 4) The keywords should be included in the title/abstract of the article.

Strategy. In the present study, the following databases were used for searching articles: Cochrane Library, PubMed, CINAHL, Science Direct, Wiley Scopus, Request, Web of Science, Elsevier, Google Scholar, and Ovid. Research was performed using the search operators OR and AND. In addition, the keyword control "Mesh", which is available in PubMed, was used to find words related to the subject of the study. The following keywords were used individually and in combination for the search: Competence, qualification, ability, authority, autism, children with autism, parents, caregiver, parenting competence, parents with autistic children, and parental competence scale.

Selection of articles. Initially, three of the researchers individually made a list of the titles and abstracts of all the articles collected from the databases. Subsequently, the articles which appeared on the list more than once were omitted. Next, the abstracts were carefully reviewed and irrelevant articles were omitted. The remaining articles were thoroughly examined and evaluated. Eventually, the articles which addressed the questions raised in the present study were included in the systematic review.

Selection of articles for analysis. 353 relevant articles were found in the databases: 67 articles from Web of Science, 15 articles from Cochrane Library, 72 articles from PubMed, 32 articles from Cinahl, 75 articles from Google Scholar, 25 articles from Ovid, 21 articles from Science Direct, 22 articles from Scopus, 13 articles from Elsevier, and 11 articles from ProQuest. After a review of the titles of the articles, 101 articles were omitted due to repetition. Subsequently, 252 Papers evaluated by the title and abstract according to the inclusion and exclusion criteria, 200 papers were omitted because they assessed other concept in parent’s children with autism and did not assesse parenting competence, also full text 52 of the retrieved papers were concisely assessed, excluded 7 articles such as full text written in non-English language. Finally, 45 articles remained to be thoroughly examined and reviewed. 

## Results

**Figure 1:**
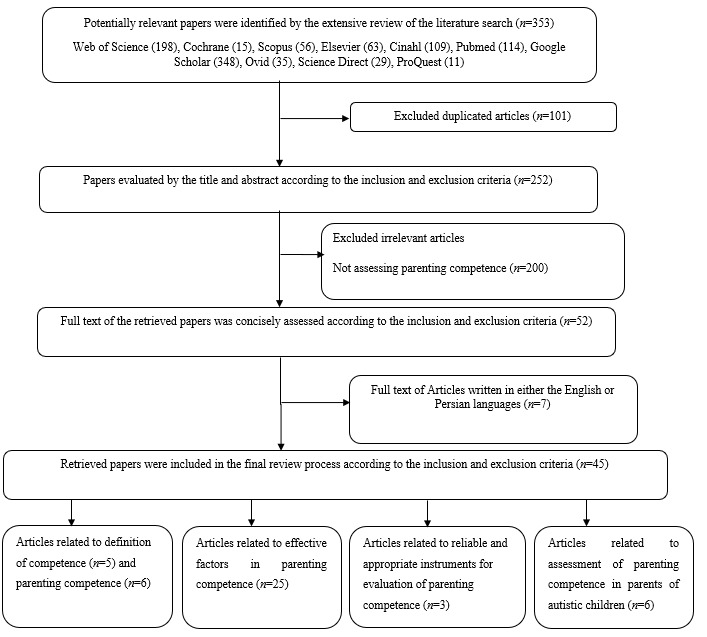
Flow diagram of the selection process for the study

Out of the 350 articles selected in the first stage only 45 dealt with the definition of parenting competence and evaluation of this concept in parents with autistic children. Among these articles, 34 were quantitative and 11 were qualitative. Additionally, 11 articles concerned the definition of competence and parenting competence, 25 involved the effective factors in parenting competence, particularly in parents of autistic children, 6 had assessed parenting competence in such parents and 3 articles developed appropriate instruments for evaluation of parenting competence. There were high risks of bias in 31 studies (mainly due to the design), moderate risks in 2 studies and weak risks in 5 studies. (Figure 1)

### What is the meaning of parenting competence?

Review of the literature indicated that competence is an expanded concept(17) with various meanings depending on backgrounds, conditions, domains, and individual characteristics.(18) Therefore, no global definition can be presented for competence.(17,18) Indeed, studies on assessment of parenting competence have provided various definitions for this concept.(11,19) For instance, Nair *et al.*(20) defined parenting competence as parents’ ability to provide care without asking others for help. In addition, Montigny *et al*.(21) conducted a study on analysis of maternal self-efficacy and stated that competence and self-efficacy were inter-related but different. Shin *et al*.(11) also disclosed that maternal sensitivity was related to, but different from maternal competence. 

From psychological point of view, competence is defined as sufficient knowledge and skills for successful and effective performance of tasks.(19,22) On the other hand, Pridham *et al*.(23) defined parenting competence as the knowledge, skills, problem solving ability, and activity for child care, with knowledge and skills being more important compared to other dimensions. Mercer(24,25) also defined maternal competence as the ability to perform maternal roles through interaction with microsystems, particularly the family. Mondell *et al*.,(26) too, defined parenting competence as self-efficacy, trust in interpersonal relationships, and compatibility with parental roles. However, no studies have been conducted on this concept in parents of autistic children.

### What factors affect parenting competence in parents with autistic children?

The results of the studies demonstrated that parenting competence could vary depending on parents’ and children’s characteristics.(27) However, it was affected by interaction with professionals,(28) acquiring skills in child care,(28,29) ability to recognize the child’s reactions,(29,30) parents’ childhood experiences,(29) mental health,(29,31) age,(29,32) self-efficacy,(33,34) and parents’ self-confidence.(23) Among these factors, parents’ self-efficacy and self-confidence were two important predictors of parenting competence.(23,35,36)

The findings of the studies on parents with autistic children revealed that stress, depression, familial and social support, parents’ mental and physical health, feeling of guilt due to child’s disease, participation in care programs, and being supported by professionals were effective in self-efficacy and subsequently parental competence of parents with autistic children.(37,38) On the other hand, other studies indicated that parents’ self-efficacy had an impact on their parenting behaviors. It is evident that parents’ behaviors and competence could, in turn, affect children’s social, emotional, and educational growth and development.(2,32,39) Accordingly, parents with higher self-efficacy showed more responsibility, kindness, sympathy, and attempt for developing appropriate behaviors in their children and, as a result, were more competent to take care of their children.(40-42)

### What are appropriate and reliable instruments for the evaluation of parenting competence?

To evaluate each concept, it is essential that there be a valid instrument(43) Since self-efficacy is one of the main predictors of parenting competence,(23,35,36) parental self-efficacy questionnaires were used to assess parenting competence in most studies.(30,43,44) However, parenting competence is rather wider in comparison to parental self-efficacy(21) and cannot be judged by the mere assessment of self-efficacy. The findings revealed three main scales for the evaluation of parenting competence, namely Parenting Sense of Competence Scale, Sense of Competence Scale of the Parenting Stress Index and parental competence scale in parents of children with autism.(40,45,46) Gibaud-Wallston *et al.*(40) designed Parenting Sense of Competence Scale in 1977. This scale contains two dimensions, namely self-efficacy, knowledge, and skills (8 items); and satisfaction, comfort, and worthiness (9 items). The items can be answered through a 6-point Likert scale ranging from completely agrees to completely disagree. It should be noted that all items receive equal scores, and higher scores represent higher competence and self-confidence. This scale was first used in 100 parents to 10-week-old neonates born through natural vaginal delivery. The reliability coefficient was reported to be 0.80 for the whole scale and 0.69 and 0.80 for self-efficacy and satisfaction dimensions, respectively.

Abidin *et al.*(45)designed Sense of Competence Scale of the Parenting Stress Index in 1990. This scale consists of 120 5-option questions including 101 main and 19 voluntary items. The items are further divided into three categories: childhood (6 items), parental (7 items), and life stress (1 item). Childhood scales include adaptability (11 items), receptivity (7 items), eagerness (9 items), creativity (5 items), inattention and hyperactivity (9 items), and empowerment (6 items). Besides, parental scales include depression (9 items), attachment (7 items), parental roles limitations (7 items), feeling of competence (13 items), social isolation (6 items), relationship with spouse (7 items), and parents’ health (5 items). Finally, life stress as the voluntary scale contains 19 items. Scores higher than the 75th percentile represent more problems and higher stress levels.

Mohammadi *et al.*(2) designed mixed-method study that developed and psychometrically of parental competence scale in parents of children with autism in 2018. This scale has two dimensions including: adapting with the present situation (9 items) and excellence in care (16 items). The parental competence scale demonstrated acceptable psychometric properties. Therefore, this scale can be used for assessing parental competence in parents of children with autism.(46)

### What instruments have been used to evaluate parental competence in parents of children with autism?

According to the results, seven articles had evaluated parenting competence in parents with autistic children using Parenting Sense of Competence Scale and Sense of Competence Scale of the Parenting Stress Index.(43,47-51) The findings of these studies indicated that autistic children’s behavioral problems affected the parents’ satisfaction regarding parental roles, self-efficacy, social interactions, fatigue, and parenting competence. However, none of these factors could predict parents’ satisfaction level, self-efficacy, and parenting competence (Table 2).

**Table 2:** Studies conducted on evaluation of parenting competence in parents with autistic children

Reference	Description
43	***Scale:* Parenting Sense of Competence Scale (PSOC)** ***Authors, year:*** Sarabi Jamab *et al.*, 2011 ***Sample characteristics:*** 21 mothers of preschool children with autism (aged 3-7 years) ***Components of the Measure:*** 16 items; general measure ***Psychometrics:*** Cronbach’s α=0.57 ***Results:*** Parents training caused no increase in their self-efficacy, satisfaction, and parenting competence.
47	Scales: The Parenting Stress Index (PSI) *Authors, year:* Baker-Ericzen *et al.*, 2005 *Sample characteristics:* parents of 37 children with Autism Spectrum Disorders (ASD) and 23 typically developing children (TDC). *Components of the Measure:* 120 items; domain-specific measure; The Child Domain, The Parent domain. *Psychometrics:* Cronbach’s α=0.92. *Results:* Parents with autistic children reported higher stress levels compared to those with normal children. Nevertheless, mothers with autistic children showed a significant reduction in their stress in relation to their children, not parenting stress. They also noted that children’s social skills were the most important factor affecting their stress levels. However, no significant difference was observed in fathers’ stress levels in child-related and parenting dimensions.
48	Scale: The World Health Organization Quality of Life Assessment-BREF self-administered instrument (WHOQOL-BREF) *Authors, year:* Dardas *et al.*, 2014. *Sample characteristics:* 184 parents of children with Autistic Disorder. *Components of the Measure:* 26 items; domain-specific measure; (physical, psychological, social, and environmental health) and two individual items about an individual’s overall perception of QOL and health. *Psychometrics:* Cronbach’s α=0.67-0.93. *Results:* The results showed no significant difference between mothers and fathers with autistic children regarding physical, mental, social, and environmental health. Moreover, parents’ quality of life was significantly associated with stress, adaptation strategies, and demographic features.
49	Scale: Parenting Sense of Competence Scale (PSOC) *Authors, year:* Giallo *et al.*, 2011 *Sample characteristics:* 59 Parents of children with ASDs aged between 2 and 5 years *Components of the Measure:* 16 items; general measure. *Psychometrics:* Cronbach’s α=0.75. *Results:* Mothers of autistic children reported higher fatigue levels compared to those with normal children. Additionally, mothers’ fatigue was associated with age, occupation, and number of autistic children in the family. On the other hand, a significant relationship was found between parents’ self-efficacy and fatigue, depression, anxiety, low-quality sleep, and need for social support. However, none of these factors could predict satisfaction level and self-efficacy in parents with autistic children.
50	Scales: Parenting Sense of Competence Scale (PSOC) *Authors, year:* Rezendes *et al*., 2011 *Sample characteristics:* 140 mothers of children between the ages of 3 and 16 with Autism Spectrum Disorders (ASDs) *Components of the Measure:* 16 items; general measure. *Psychometrics:* Cronbach’s α=0.69. *Results:* Parenting stress was affected by the relationship between children’s behavioral problems and parents’ self-efficacy. On the other hand, parents’ self-efficacy was associated with the relationship between parental stress and depression and anxiety. Accordingly, behavioral problems increased parents’ stress levels, decreased their self-efficacy, and increased their anxiety and depression.
51	Scales: Parenting Sense of Competence Scale (PSOC) *Authors, year:* Rodriguez *et al.*,1992 *Sample characteristics:* Fathers of 20 autistic, 20 Down syndrome, and 20 developmentally normal children *Components of the Measure:* 16 items; general measure *Psychometrics:* Cronbach’s α=0.69. *Results:* The results revealed a significant difference between the fathers of children with autism and Down syndrome and those with normal children regarding familial function and interpersonal relationships. On the other hand, fathers of children with autism and Down syndrome mentioned that they repeatedly used information seeking and fantasizing as defense mechanisms.

## Discussion

The present study aims to define parenting competence and determine an appropriate instrument for its evaluation among parents with autistic children. The results indicated that competence was quite an expanded concept depending on backgrounds, conditions, and individual characteristics. Therefore, no global definition could be provided for this concept.(17,18) However, the results indicated that a few studies have explained parenting competence in parents with normal children(11,19-21,23-26) These studies have defined parenting competence as skills, knowledge, comfort, and satisfaction regarding the successful and effective performance of parenting roles.(19,22) However, parental competence is not defined for the parents of children with autism.

This study has also demonstrated that more factors affected parental competence in parents with autistic children, compared to those with normal children, because behavioral problems children with autism cause their parents to experiencing more.(23,52,53) Parents’ stress, anxiety, depression and fatigue levels were increased by behavioral problems, whereas their self-efficacy was decreased.(40,54) Therefore, acquiring child care skills and the ability to recognize children’s reactions play a critical role in promoting parenting competence.(29,30) Evidence has revealed self-efficacy, self-confidence, participation in care, and professional relations as predictors of parenting competence.(23,35,36) Accordingly, parents’ self-efficacy affects their parental behaviors, and this improved behavior influences their parenting competence as well as their children’s growth and development.(55) Indeed, parents’ participation in child development programs plays a key role in the promotion of parenting competence, because the parents acquire skills for organizing children’s behaviors and benefit from professional support.(56,57) Since self-efficacy is one of the main predictors of parenting competence, most studies had utilized parental self-efficacy questionnaires in order to assess parenting competence.(29,44)

The study results indicated that some studies involved designing instruments for the evaluation of parental self-efficacy in parents of children with other diseases. However, parenting competence has a far wider definition compared to parental self-efficacy.(21) Thus, parenting competence cannot be judged by the mere assessment of parents’ self-efficacy, Therefore this study identified only three scales are available in this regards, namely Parenting Sense of Competence Scale, Sense of Competence Scale of the Parenting Stress Index and parental competence scale in parents of children with autism.(40,45,58) Parenting Sense of Competence Scale is a general instrument for determining parenting competence in parents with normal children.(59) Sense of Competence Scale of the Parenting Stress Index is one sub scale with 13 items for evaluating parenting competence.(59) Although this questionnaire is a reliable scale for assessing parental stress, it is not comprehensive enough to be utilized for the evaluation of parental competence.(45) Review of the related literature revealed that parental competence scale in parents of children with autism is only specialized instrument for the assessment of parenting competence in parents of children with autism. This scale was designed in 20019; therefore this scale has not been used by other authors.(57) Nonetheless, 7 articles were found to evaluate parental competence in parents of children with autism, in which they had used these two non-specialized instruments to determine parenting competence and self-efficacy in parents of children with autism. The results of these seven studies with those of the two non-specialized scales for the parents of children with autism revealed that parents’ quality of life was significantly associated with stress, adaptation strategies, and demographic features, but it showed no significant difference between mothers and fathers of autistic children regarding physical, mental, and social health.(60) 

One of the limitations of the present study was that the Literature about explaining the concept of parental competence of children with autism and its standard tools are few, the reason for its scarcity can be the absence of a comprehensive definition of the concept of parental competence and the lack of an appropriate scale for its measurement. Furthermore, most of the studies found on the subject were carried out in developed countries. Since the level of health care and services in developed countries is higher and social welfare is generally better compared to developing countries, the findings cannot be generalized to developing countries (such as Iran). 

## Conclusion.

Although parental competence has been proposed as the main element in improving the quality of life among parents with autistic children, there have been few empirical studies and research; however, as parental competence is one of the key features among parents, it should be defined in parents with autistic children. An appropriate instrument should also be developed to evaluate parenting competence in such parents. The findings of the present study can develop the knowledge and attitude of researchers and healthcare providers regarding the evaluation of parenting competence of parents with autistic children and subsequently, improve families’ and children’s health status. Thus, based on our findings, it can be said that the development and definition of parental competence concept among parents of autistic children, as well as the promotion and development of assessing the parental competence will be an important aspect of nursing care and clinical performance. Therefore, the development of this concept is highly essential for clinical application and investigating its outcomes support. In order to understand parental competence of parents with autistic children, it is suggested that mixed research be used to define this concept, then development and psychometric scale for assessing parental competence of such parents in different cultures. 
